# Coarse-Grained Monte Carlo Simulations of Graphene-Enhanced Geopolymer Nanocomposite Nucleation

**DOI:** 10.3390/nano15040289

**Published:** 2025-02-13

**Authors:** Mohammadreza Izadifar, Neven Ukrainczyk, Eduardus Koenders

**Affiliations:** Institute of Construction and Building Materials, Technical University of Darmstadt, Franziska-Braun-Str. 3, 64287 Darmstadt, Germany; koenders@wib.tu-darmstadt.de

**Keywords:** geopolymer, 3D off-lattice, coarse-grained Monte Carlo simulations, graphene-reinforced geopolymer nanocomposite, pH, nucleation, graphene-based nanomaterials

## Abstract

Geopolymer nanocomposites, incorporating pristine graphene-based nanomaterials, are at the forefront of research in advanced construction materials, improving mechanical, electrical, and thermal properties. This study investigates the nucleation mechanisms of geopolymers on pristine graphene substrates, namely graphene-reinforced geopolymer nanocomposites (GRGNs), by analyzing nanostructure particle sizes, pore size distributions, cluster sizes, and system energy at a pH of 11, compared to a system without graphene nanosheets. Seven distinct monomer species were selected to observe cluster evolution over numerous iterations, providing insights into the dynamic nature of geopolymer nucleation on graphene-based substrates. Thus, the computed adsorption energies, based on recent DFT studies, reveal interactions between aluminosilicate species and graphene nanomaterials. Furthermore, the implementation of energy values from dimerization reactions among monomer species, as reported earlier, introduces tetrahedral geometrical constraints, crucial for understanding how particles aggregate into clusters. The key findings indicated that (4.34%) fewer particles participate in cluster formation in the system containing a graphene nanosheet compared to the one without it. However, the system with the graphene nanosheet exhibits (1.65%) more favorable energy. This contrast is due to the weaker adsorption energy on the graphene nanosheet (heterogenous nucleation) than in homogenous particle nucleation. The complete dissolution of MK required (4.54%) more iterations in the system with graphene than in the system without it. This research underscores the significant potential of geopolymer nanocomposites and their role in shaping the future of construction materials.

## 1. Introduction

The word “geopolymer”, introduced by Davidovits in 1978 [1], denotes materials formed by combining aluminosilicate powder with a precursor solution of potassium silicate or sodium silicate [2,3]. The initiation of the alkalinization process is significantly influenced by silicate solution precursors. In the dissolution–precipitation geopolymerization reaction, hydroxyl (OH^−^) groups in the alkaline solution first break the bonds between silicate and aluminate in the solid aluminosilicate powder material (metakaolin, MK) [4,5]. This is followed by a polycondensation reaction that leads to the creation of an aluminosilicate network [6]. This network comprises interconnected aluminate and silicate (Si) tetrahedra connected by oxygen bridging bonds [3]. Geopolymer binders demonstrate mechanical properties comparable to those of ordinary Portland cement, yet their production generates roughly 80–90% fewer global anthropogenic CO_2_ emissions [7,8]. Tailby and Mackenzie [9] reported that the compressive strength of pure sodium geopolymer after twenty-eight days ranges between 67 and 89 MPa. In contrast, ordinary Portland cement exhibits a compressive strength of 29 to 43 MPa, highlighting the superior mechanical performance of geopolymers. This characteristic establishes geopolymers as a sustainable and eco-friendly substitute for conventional cement [10]. The core chemical and structural properties of geopolymers produced from MK [11], slag, and fly ash have been analyzed to evaluate how the selection of raw materials impacts the characteristics of the resulting geopolymer composites [3]. Geopolymers exhibit superior resistance to acid compared to Portland cement, a characteristic frequently highlighted as one of the key advantages of geopolymers [12,13,14]. Additionally, geopolymers possess inherent fire resistance and exhibit exceptional thermal stability, surpassing that of conventional cements [15].

The nanoscale modeling of geopolymers has recently attracted significant interest among researchers [16,17]. Lolli et al. [18] developed an innovative framework for understanding the molecular structure of geopolymers, highlighting the significance of nanoscale interfaces between crystalline and amorphous regions in influencing the materials’ mechanical behavior. Izadifar et al. [19] studied the polymerization mechanisms of alkaline aluminosilicate gels using a three-dimensional off-lattice coarse-grained Monte Carlo (CGMC) method [20], which also enabled the characterization of their nanostructural features, such as particle size distributions and pore size distributions. The input data for this analysis included the Gibbs free energy values of the dimerization reactions for four different monomer species, obtained from the literature and computed using the DFT modeling approach, as described by White et al. [10,21]. Moreover, Valencia et al. [22] have lately employed the CGMC simulation method with Octree cells for geopolymer nucleation [23] at different pH values. Yang and White [24] employed alkali-activated materials (AAMs), specifically class F fly ash and MK, at the mesoscale using the CGMC simulation method combined with the DFT computational method. Their findings revealed that in both H-activated fly ash and MK systems, the growth of the gel occurred via the development of medium-sized clusters in conjunction with the expansion of the largest particle. Subsequent to this process, certain small clusters persisted in the pore solution of the solidified gel, with their dimensions determined by the saturation level of pre-dissolved silicate concentrations within the system. Furthermore, Izadifar et al. [4,5] have recently calculated the enthalpy activation energy (Δ*H*^∗^) under far-from-equilibrium conditions, utilizing the transition state theory (TST) to determine atomistic reaction rates for silicate tetrahedra dissolution in MK via a (DFT) computational approach [25]. White et al. [26] also employed quantum chemical-based interaction energies, computed via density functional theory computations. They utilized an on-lattice CGMC simulation to investigate the primary stages of gel/cluster formation in sodium silicate systems across various concentrations. Their model, implemented on a cubic lattice comprising 125,000 sites in the canonical ensemble (NVT) with periodic boundary conditions, facilitated the assessment of structure evolution resulting from polymerization reactions through Monte Carlo moves and total system energy minimization.

Materials with two-dimensional structures, like graphene [27,28,29,30,31,32], have gained remarkable attention in nanomaterials study because of their exceptional thermal [33,34,35,36,37], mechanical [38,39], and electrical properties [40,41]. GRGNs have been explored across various advanced material applications, including structural health monitoring, structural supercapacitors, hydrogen production, 3D printing, dye wastewater treatment, and concrete, showing promising outcomes [42]. Gao et al. [43] showed that CNT-based cementitious composite outperforms CF counterparts, specifically exhibiting significant advancements in thermoelectricity by 160% and compressive strength by 269%. Moreover, the high length-to-diameter ratio of CNTs contributes to their improved electron transport efficiency, consequently enhancing the thermoelectric properties (Seebeck effect) and piezoelectric properties (piezoelectric effect) of cementitious materials. Zhang et al. [44] synthesized a new graphene-bottom-ash-based geopolymer (GBAG) composite using alkaline-activated geopolymerization. The primary aim was to enhance the electrical conductivity of the GBAG by integrating graphene into its otherwise non-conductive matrix. Zhong et al. [45] for the first time reported the extrusion-based 3D printing of geopolymer/GO nanocomposite structures. Their study showed that incorporating GO significantly alters the rheological behavior of the geopolymer precursor, facilitating the 3D printing of structures with a GO/geopolymer (GOGP) blend, a capability not achievable with pure geopolymers. We clarify the research gap by emphasizing the need for a more detailed understanding of the nucleation process of alkaline aluminosilicate gels in the presence and absence of graphene nanosheets through a nanoscale modeling approach. The aim of this study is to focus on the geopolymerization reaction process, comparing systems with and without graphene nanosheets to evaluate their influence on adsorption interactions with monomer species, participating in cluster formation and energy stabilization. Thus, we used 3D off-lattice CGMC simulation method, both with a GRGN and without a pristine graphene nanosheet, to investigate the nucleation mechanism of alkaline aluminosilicate gel in a silicate-activated system. The Gibbs free energy associated with the process of dimerization, derived from a study by White et al. [10], was used for four monomer species of Si(OH)_4_, Al(OH)_4_^−^·Na^+^, SiO(OH)_3_^−^·Na^+^·3H_2_O, and SiO_2_(OH)_2_^2−^·2Na^+^·6H_2_O. Furthermore, we incorporated the adsorption energy (E_ad_), including the van der Waals (vdW) interactions between a pristine graphene nanosheet and four monomer species, as detailed in Table 1 of the recent study by Izadifar et al. [46], to investigate the nucleation process in this study. Each monomer type is modeled as a separate coarse-grained particle type to investigate the evolution of gel structure across varying numbers of iterations. We explore the pH value of 11, employing an expanded simulation system with Octree cell expansion to enhance accuracy. The system’s total energy is calculated at various iterations, offering valuable insights into its behavior. Moreover, we analyze the formation of clusters and the dissolution of MK over 56,000,000 iterations. The final structure is analyzed to evaluate cluster size distribution and pore network features.

## 2. Simulation Model and Method

### 2.1. Atomistic Model Preparation

The off-lattice CGMC method began by defining the number of particles for the four different monomer species, based on the selected activated solution and pH level. For the chosen silicate-activated system, the contributions of water as well as Na (68.4%), silicate in solution (10.6%), and MK (21.0%) were acquired from the data (shown in Table 3) provided by White et al. [10]. For this, a cubic simulation box with a size of 100 Å was selected, and the percentage of each type of monomer species was computed. For the silicate-activated system, the contribution of silicate in the solution and MK were equivalent to the simulation boxes with dimensions of 47.32, and 59.43 Å, respectively. Hence, the total count of silicate monomers in solution and MK particles (in a simplified crystal arrangement) within the simulation box, with average diameters of 3.3 and 4 Å, were determined as 2744 and 2744, respectively. Given the three types of silicate monomers in solution, namely Si(OH)_4_, SiO(OH)_3_^−^·Na^+^·3H_2_O, SiO_2_(OH)_2_^2−^·2Na^+^·6H_2_O, the distribution percentages of these three contributed silicate monomer types (2744 particles in total) to maintain the pH at 11 were computed as 5%, 90%, and 5%, respectively [47]. These percentages translate to particle numbers of 137, 2470, and 137 (2744 particles in total), respectively, as per Figure 5 published by Sefcik and McCormick [47]. Furthermore, the proportion of particles in MK present as alumina (aluminum oxide) and silica (silicon dioxide) were determined to be 42% (1152 particles) and 58% (1592 particles), respectively [10]. Moreover, the adsorption energy (E_ad_) incorporating the van der Waals (vdW) dispersion forces between a pristine graphene nanosheet and the four monomer species of Si(OH)_4_, Al(OH)_4_^−^·Na^+^, SiO(OH)_3_^−^·Na^+^·3H_2_O, and SiO_2_(OH)_2_^2−^·2Na^+^·6H_2_O was extracted from Table 1 reported by Izadifar et al. [46].

### 2.2. Monte Carlo Approach: Implementation in MATLAB Code

**Figure 1:**
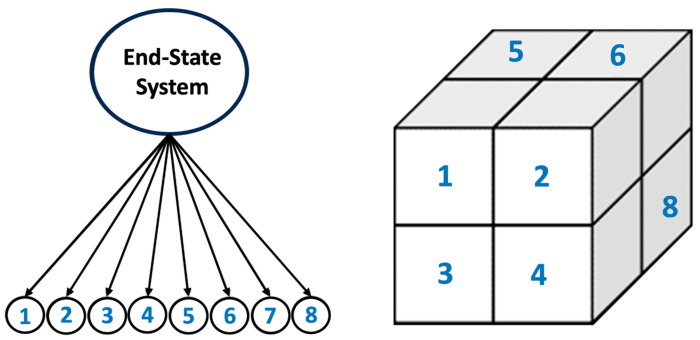
The system is split into eight regions, with each region representing a subsystem, and arranged in an Octree configuration.

**Figure 2:**
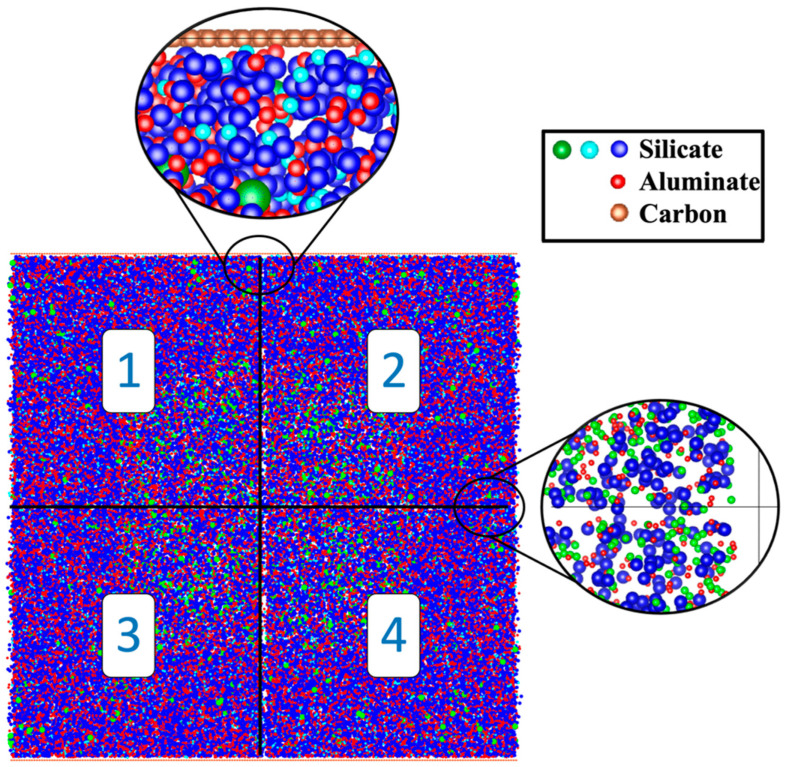
The pH11 system is depicted in a side view, providing a close-up of the interface planes within the Octree simulation cell structure. Particles positioned at the boundaries of each subsystem are connected to adjacent cells, enhancing the overall interconnectivity of the system.

**Figure 3:**
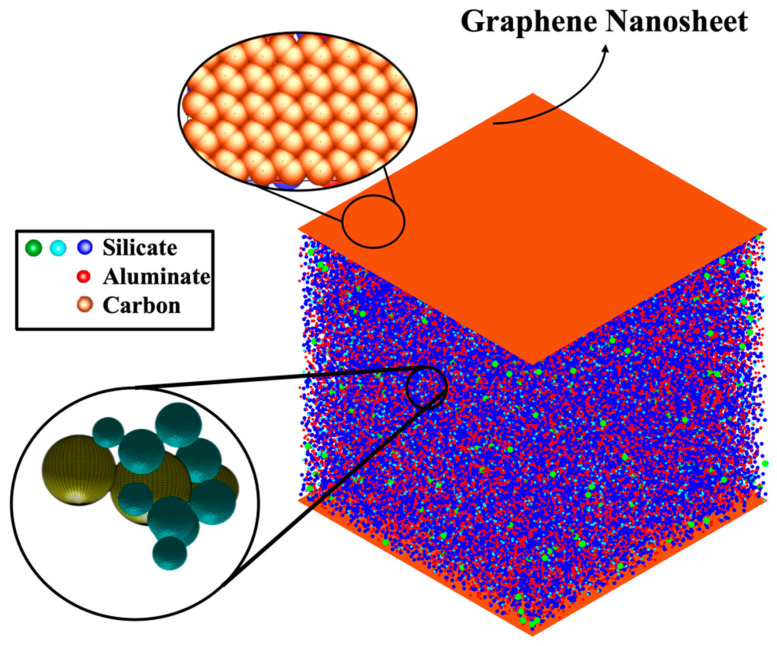
The concluding simulation snapshot at pH 11 displays clusters, particles, and pore distribution on the right side. On the left side, the calculation of pore size distribution is detailed: dark aquamarine-colored particles represent coarse-grained monomer particles (without distinguishing types), while yellow particles indicate pore sizes.

Custom MATLAB code [48,49] was created to manage and analyze data within exclusive cells following an Octree pattern, as depicted in Figure 1. To include data from adjacent cells, a neighbor relationship was defined, where two sites were considered neighbors only if they shared a common face, adjacency via an edge or corner not being sufficient. This methodology ensured that all interactions between clusters and monomers across cells were captured. It is also worth mentioning that all the reported numbers from Section 2.1 concerning silicate monomers in solution, as well as silica and alumina particles from MK, had to be divided by 8 partitions, each representing a subsystem according to an Octree pattern. Figure 2 provides a detailed side view where two subsystems share particles involved in the geopolymerization process. The software tracked detailed information during the simulation, covering monomer specifications, cluster dynamics, system energy fluctuations, and the complex MK dissolution process. Post-simulation analysis involved using the global scan method to extract cluster size distribution. A refined pore network model was constructed, assuming idealized spherical pores, with monomers and dimers considered as aqueous species, as reported by White et al. [10]. Our particle structure digitization utilized a watershed algorithm and a city-block distance transform function (Figure 3). Subsequently, the analysis of pore connectivity and size distribution was conducted based on the approach detailed by Izadifar et al. [19]. The system’s overall energy was evaluated using the Gibbs free energy associated with dimerization, as documented by White et al. [10].

### 2.3. Octree Cells Approach: MATLAB Program Development

Models based on atomistic theory often face challenges in capturing mesoscale phenomena in zeolites due to the high computational costs involved in simulating across the necessary length scales [2]. The principal aim was to explore particle behavior during polymerization processes by employing a simplified mechanical model. This investigation is essential for understanding nanoparticle formation, as larger system sizes are required to produce statistically meaningful results. Furthermore, simplified models enable a more concentrated examination of particular system characteristics [50]. The simulation methodology used in this study builds upon our former research [19], with a system size of 100 × 100 × 100 Å^3^. To facilitate this, an Octree cell structure with an initial partitioning level was developed, enabling parallel simulation, which is commonly utilized in high-performance computing (HPC) environments. Specifically, eight identical simulations were executed simultaneously, each with 7,000,000 iterations, and at the end, their results were combined for a thorough structural analysis (as detailed in Section 2.2). The use of Octree patterns considerably lowered the memory demands for the CGMC solver when simulating large-scale systems, balancing the reduction in artificial effects in smaller lattice simulations with achieving convergence in a rational time frame [22].

### 2.4. Density Functional Theory (DFT) Computational Modeling Method

**Figure 4:**
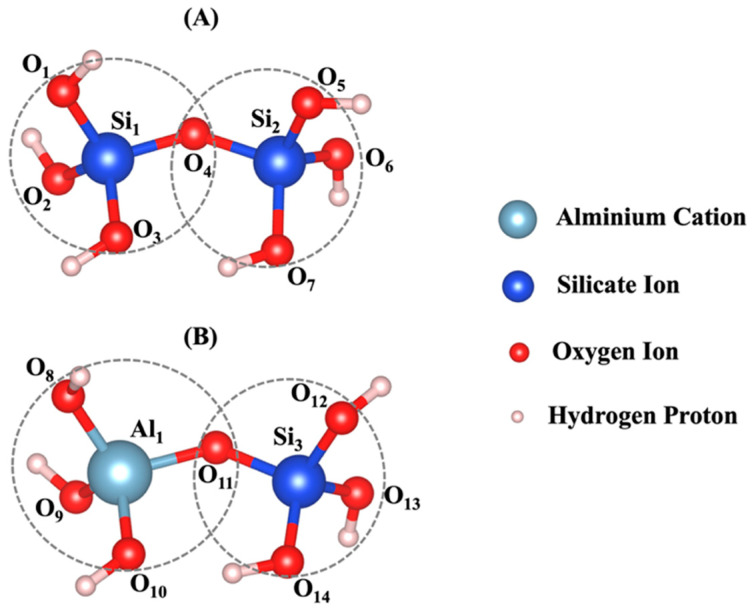
The dimerization reaction, characterized by tetrahedral formation for (**A**) Si-O-Si and (**B**) Si-O-Al, was optimized using the DFT computational modeling method. The radius of the coarse-grained particles was determined based on the average bond length. Interaction between the two particles occurs at a single point, located at the center of the bonding oxygen atom O4 for case A and O11 for case B.

The density functional theory (DFT) [51] computational modeling method was conducted to determine the geometrical parameters of the silicate and aluminate monomers (Figure 4). The Vienna ab initio simulation package (VASP) [52,53,54,55,56] was utilized, employing the projected-augmented wave (PAW) method [57] and pseudopotential to model electron–ion interaction. The electron exchange and correlation energy were selected based on the generalized gradient approximation (GGA) with Perdew−Burke−Ernzerhof (PBE) parametrization [58]. The Brillouin zone was sampled using a well-converged k-sampling equivalent, given by a 1 × 1 × 1 Monkhorst–Pack k-points mesh size for the entire system [59]. A plane–wave cutoff energy of 400 eV was employed for structural relaxations, ensuring convergence. The electronic self-consistent cycle convergence criterion was set at 10^−6^ eV, while ion relaxation continued until forces fell below 10^−3^ eV/Å. Additionally, structural analysis was carried out using the three-dimensional visualization software VESTA [60].

## 3. Results and Discussions

**Figure 5:**
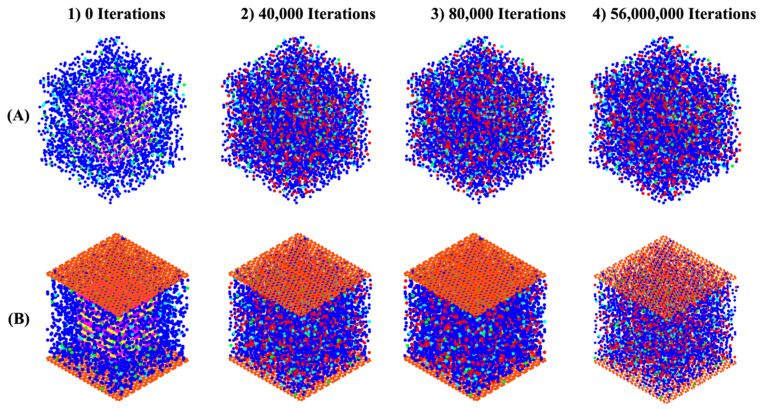
The progression of structures and cluster formation in geopolymer systems (**A**) without and (**B**) with the contribution of a graphene nanosheet are observed at specific iteration points: 0, 40,000, 80,000, and 56,000,000. The addition of MK into the system is indicated at iteration 0, preceded by pre-equilibration occurring from iteration 8,000,000 to iteration 0.

**Figure 6:**
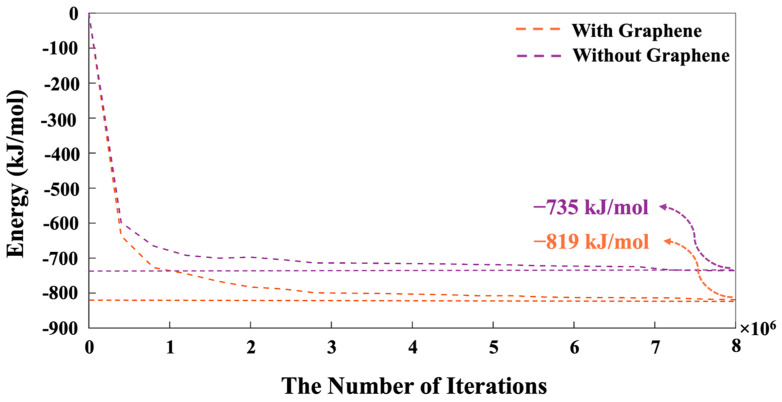
The energy progression of silicate species (particles), both with and without the influence of a graphene nanosheet, after 8 million iterations in solution at pH 11, before the introduction of MK.

The distances between bonded atoms and the angles formed by these bonds, computed through DFT calculations, are detailed in Table A1-2 from our recent study, respectively [22]. Based on tetrahedral formation, we calculated β angles of 135.12° and 138.28° for the dimerization reactions of Si3-O11-Al1 and Si1-O4-Si2 (as shown in Figure 4), respectively. For the Si-O and Al-O bonds in tetrahedral monomer formation, we considered mean values of bond lengths of 1.65 and 1.76 Å, respectively, which define the radius of coarse-grained particles specific to each particle type. Additionally, mean angles of 108° and 107° were assumed for O-Al-O and O-Si-O angles in the formation of tetrahedral monomers, respectively, specifying each particle pairing type. The process of polycondensation brings the monomers together, leading them to connect at a single point—the shared center of the bonding oxygen between both particles (as illustrated in Figure 4). At pH 11, images capturing the evolution of the two geopolymer systems, including with and without the contribution of graphene nanosheet, at a certain number of iterations of 0, 40,000, 80,000, and 56,000,000, are observed from Figure 5A,B, respectively. The coarse-grained particles representing various monomer building units are color-coded as follows: Si(OH)_4_ particles are depicted in cyan, SiO(OH)_3_^−^·Na^+^·3H_2_O particles in blue, SiO2(OH)_2_^2−^·2Na^+^·6H_2_O particles in green, and Al(OH)_4_^−^·Na^+^ particles in red. The final equilibrium condition was reached after 56 million iterations. The percentage of total particles involved in gel formation (cluster formation) was found to be 59.48% without the contribution of a graphene nanosheet and 57.27% with the contribution of a graphene nanosheet. Figure 6 depicts the equilibrium conditions observed during the energy computation for the silicate-activated system at pH 11, both with and without the contribution of a graphene nanosheet, after 8 million iterations within the solution, excluding MK.

**Figure 7:**
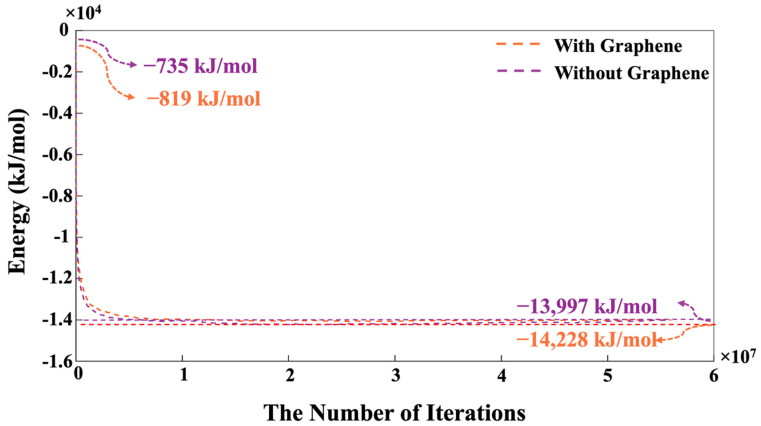
During 56 million iterations and with the involvement of MK, the equilibrium condition for the pH 11 system was determined by performing energy minimization calculations with and without the contribution of a graphene nanosheet. The introduction of MK into the system is indicated at iteration 0, followed by a pre-equilibration period lasting an additional 8,000,000 iterations (Figure 6).

In more detail, the system’s energy decreased sharply during the first 500,000 iterations (more favorable lower energy state). Between 500,000 and 1,000,000 iterations, the decline continued at a lower slope, after which the energy gradually stabilized, eventually reaching an equilibrium condition. Indeed, this pre-equilibrium condition was conducted for dissolved activator silicates. It is notable that the system containing pristine graphene nanosheet exhibited a lower energy of −819 kJ/mol compared to the system without the contribution of a graphene nanosheet, indicating a more favorable condition. As depicted in Figure 7, the moment when MK is introduced into the system is marked as iteration 0. In other words, the pre-equilibration condition obtained from iteration 8,000,000 (as shown in Figure 6) has been set to iteration 0 with the MK involvement, as shown in Figure 7. Therefore, the system’s energy at the simulation’s outset is not zero, indicating the pre-equilibration process, and the involvement of the silicate monomers in the solution as activators is examined.

**Figure 8:**
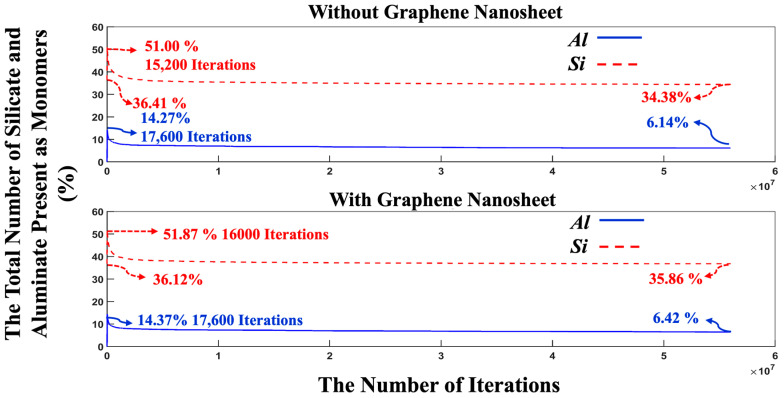
The variation in the quantity of silicate and aluminate monomers within the simulated system is monitored over 56,000,000 iterations for the pH 11 system, both with and without the involvement of a graphene nanosheet. MK particles are regarded as monomers solely during the dissolution process.

Figure 7 indicates a swift reduction in energy, implying the dissolution of MK particles and the initiation of the polymerization process, where the aluminum monomers from the MK particles play a crucial role. After 500,000 iterations, the energy trend begins to stabilize, and by 56 million iterations, the energy values for the systems, both with and without the presence of the graphene nanosheet converge completely to values of −13,997 kJ/mol and −14,228 kJ/mol, respectively. The energy evolution for the pre-equilibrium condition after 8 million iterations (Figure 6) and total system energy over 56 million iterations (Figure 7) confirm that the graphene-containing system maintains a lower energy (more favorable energy) due to the adsorption interactions between monomer species, participating in cluster formation with the graphene nanosheet. Figure 8 depicts the variation in the percentage of aluminate and silicate monomers for two systems, one with graphene contribution and one without, at pH 11 throughout the simulation. At the initiation of MK dissolution, the concentration of aluminate monomers remains at zero for both systems, with and without the contribution of a graphene nanosheet, as all aluminate particles are initially contained within the MK particle. In contrast, the total concentrations of silicate monomers present in both systems from the pre-equilibrium condition at 8 million iterations are 36.41% (without graphene nanosheet) and 36.12% (with graphene nanosheet). These starting values represent the quantity of silicate monomers within the solution. Over time, the concentrations of aluminate and silicate monomers rise significantly due to the dissolution of the precursors. The highest amounts of silicate monomers in the solution are 51.87% at 16,000 iterations (with graphene nanosheet) and 51.00% (without graphene nanosheet) at 15,200 iterations.

**Figure 9:**
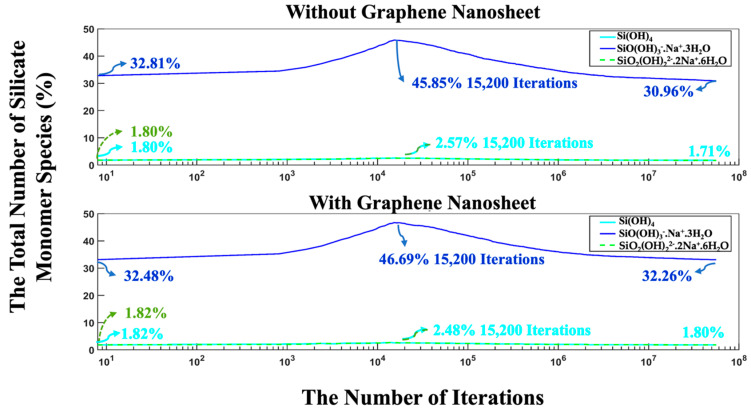
During 56 million iterations, the percentage of silicate monomers within the system is tracked, with and without the incorporation of a graphene nanosheet. At the beginning of the simulation (iteration zero), MK species (particles) were excluded as they dissolved during the process.

Moreover, the highest amounts of aluminate presented as monomers in the solution reach 14.37% (with graphene nanosheet) and 14.27% (without graphene nanosheet) at 17,600 iterations. After 56 million iterations, the number of monomers in the solution has decreased due to cluster formation. It was observed that the number of remaining aluminate and silicate monomers in the solution was fewer in the system without a graphene nanosheet compared to the system with a graphene nanosheet. To enhance the comprehension of the varying silicate contributions to cluster formation over time, Figure 9 illustrates the total number of silicate monomer species over different iterations. The three different types of monomer species, namely Si(OH)_4_, SiO(OH)_3_^−^·Na^+^·3H_2_O, and SiO_2_(OH)_2_^2−^·2Na^+^·6H_2_O, contributed 1.80, 32.81, and 1.80% (total 36.41%, as depicted in Figure 8) at the beginning of the simulation, respectively, for the system without a graphene nanosheet. In the case of graphene nanosheet contribution, the same previous monomer species contributed 1.82, 32.48, and 1.82% (total 36.12%, as depicted in Figure 8), respectively. It is also worth reporting that the amount of SiO(OH)_3_^−^·Na^+^·3H_2_O in the solution after 8 million iterations in the pre-equilibrium condition and at the beginning of MK dissolution is high (32.81% for the system without graphene, and 32.48% for the system including a graphene nanosheet) due to its high contribution, equal to 90% (as explained earlier in Section 2.1) in the silicate-activated system.

The highest amount of these three silicate monomers presented at 15,200 iterations due to MK dissolution, and at the end, after 56 million iterations, the amount of SiO(OH)_3_^−^·Na^+^·3H_2_O had decreased to 30.96% and 32.26% for both systems, without and with the incorporation of a graphene nanosheet, respectively. According to Figure 7, Figure 8 and Figure 9, although the system containing the graphene nanosheet exhibits a more favorable (i.e., more negative) energy, fewer Al and Si monomer species participate in the nucleation process compared to the system without the nanosheet. This can be attributed to the adsorption energy between the graphene nanosheet and monomer species participating in cluster formation, which influences the overall energy computation.

**Figure 10:**
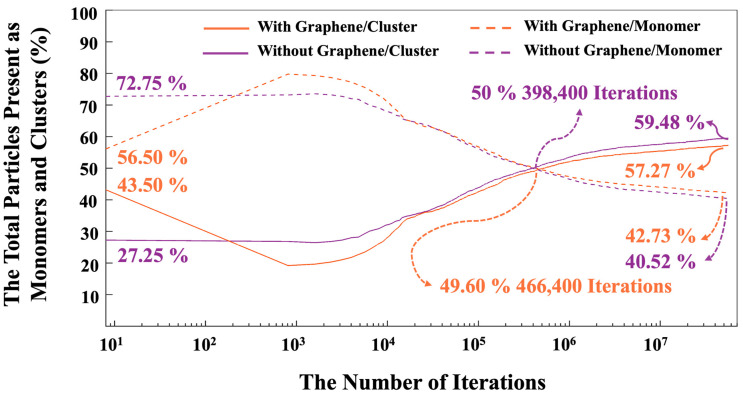
The evolution of monomer numbers, both participating and not participating in cluster formation, was tracked over 56 million iterations for both systems, one incorporating a graphene nanosheet and the other without.

Figure 10 illustrates the trend of total particles present as monomers and clusters during 56 million iterations for both systems, with and without the incorporation of a graphene nanosheet. It is essential to note that graphene particles have not been factored into the reported percentage of monomer and cluster contribution for the system including graphene a nanosheet, as the role of graphene is crucial for the adsorption of different species onto it. The point where both monomers and clusters achieved an equal percentage (50%) occurred at 466,400 iterations for the system with the graphene nanosheet and at 398,400 iterations for the system without it. After 56 million iterations, it is evident that the number of particles participating in cluster formation is (4.34%) lower in the system with graphene nanosheet (57.27% particles participated) compared to the system without it (59.48% particles participated). Conversely, the energy of the system including a graphene nanosheet is more favorable (more negative, 1.65% higher) (−14,228 kJ/mol) than the system without graphene (−13,997 kJ/mol), as depicted in Figure 7. Up to 1000 iterations, the cluster formation remained constant in the system without a graphene nanosheet, whereas it showed a decreasing trend in the system with graphene. However, after 1000 iterations, both systems exhibited an increasing trend in cluster formation. This discrepancy can be attributed to the higher adsorption energy between the graphene nanosheet and particles contributing to cluster formation.

**Figure 11:**
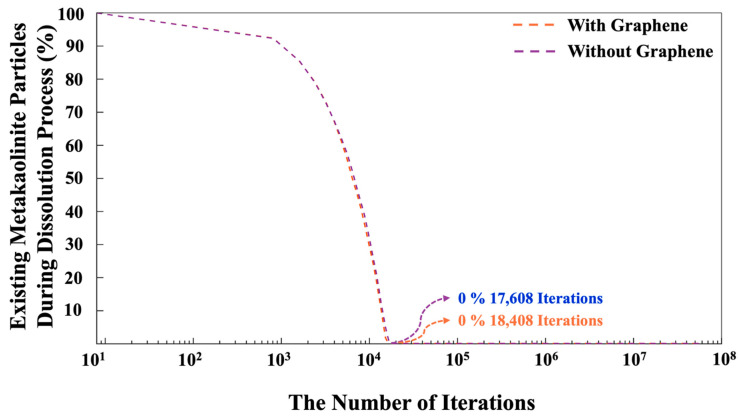
The dissolution process of MK, with and without the incorporation of a graphene nanosheet, are observed over 56 million iterations.

Figure 11 illustrates the progression of existing MK particles during the dissolution process for both systems, with and without the presence of a graphene nanosheet. The total dissolution of MK at various iterations has been observed for both systems. The main reason in the various computed iterations for MK dissolution obtained from both systems can be attributed to the fact that the MK dissolution can be accepted or rejected. After a random MK particle dissolves (either alumina or silica), the system checks for overlaps with other particles. If no overlap is detected, the particle movement is accepted within the Monte Carlo (MC) framework if the total system energy after the move is lower than the energy before the move. If the energy increases (becomes less negative), the movement may still be accepted based on the probability *X*, which is compared to a randomly selected value between 0 and 1. The probability *X* is determined using the Boltzmann factor for the change in configuration, as shown in Equation (1), where *kB* represents the Boltzmann constant, *T* is the temperature, and Δ*E* is the energy change.

(1) $$X=e^{\frac{-\Delta E}{kBT}}$$

(2) ∆E=E(after particle movement)−E(before particle movement)

**Figure 12:**
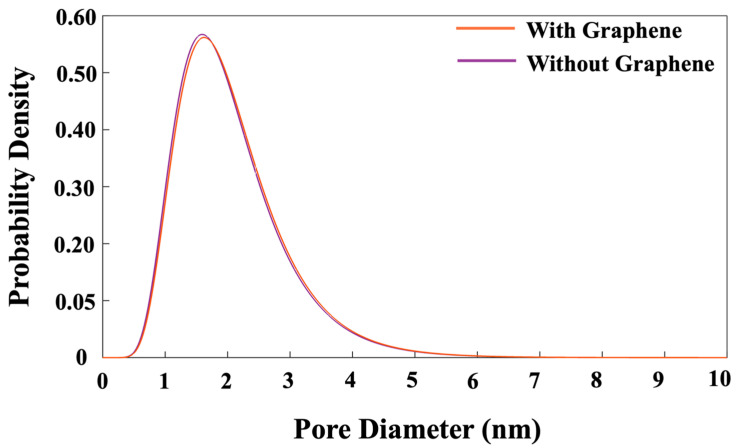
Pore size distribution after 56 million iterations for both systems with and without the incorporation of a graphene nanosheet.

If the movement is not accepted, the particle will return to its original position (MK), and the loop for the next step will begin. In the next step, a particle is randomly selected, and it is moved in any direction to a new position while ensuring that the connections between particles maintain their tetrahedral structure. The displacement of the particle is determined by generating a random number between zero and one, which is then multiplied by the radius of the moving particle. It is also worth mentioning that particles participate in cluster formation when the distance between the moved particle and its nearest neighbor is within 1 Å. The system’s total energy is then recalculated to determine whether it has increased or decreased as a result of this change, as described above. After every 20 iterations, the pH of the system is also checked to make sure to maintain the pH of the system at 11. If the pH has changed, the pH must be accordingly controlled by 5% (Si(OH)_4_), 90% (SiO(OH)_3_^−^·Na^+^·3H_2_O), and 5% (SiO_2_(OH)_2_^2−^·2Na^+^·6H_2_O) [47]. Figure 12 is also plotted to illustrate the distribution of pore sizes for both systems. The pore size distributions in both systems exhibit a similar trend. At a high probability density of 56.5%, pore diameters of 1.62 and 1.57 nm were observed for the systems with and without the incorporation of a graphene nanosheet, respectively. Due to the nucleation process, the system containing the graphene nanosheet involves a lower contribution of monomer species in the nucleation process, leading to higher porosity (pore size distribution).

## 4. Conclusions

This research explored the nucleation of aluminosilicate gel using the off-lattice coarse-grained Monte Carlo (CGMC) method, both with and without graphene nanosheets, at a system pH of 11. The tetrahedral geometry and binding energy parameters of aluminate and silicate monomers were derived from DFT simulations. To efficiently scale up the CGMC system size, a method employing the Octree cell approach was developed, significantly reducing computational time and taking full advantage of parallel high-performance computing. The results of our study can be summarized as follows:

- The Octree cell approach significantly (eight times) reduced computational time and optimized high-performance computing (HPC) resources, enabling the efficient scaling of the CGMC simulations.
- The proportion of particles involved in cluster formation was (4.34%) lower in the system with graphene compared to the one without it. In contrast, the system containing graphene displayed a more favorable energy state, which can be ascribed to the weaker adsorption energy on the graphene nanosheet (heterogenous nucleation) compared to the homogenous nucleation.
- The complete dissolution of MK required (4.54%) more iterations in the system with graphene than in the system without it, indicating slower geopolymerization due to steric hinderances and the less-favorable heterogenous nucleation.
- After 56 million iterations, the graphene-containing system exhibited a lower cluster formation percentage (57.27%), while the system without graphene showed 59.48% cluster formation.
- The system containing graphene exhibited a more favorable (1.65% lower) energy state of −14,228 kJ/mol, compared to −13,997 kJ/mol in the system without graphene. This energy difference is due to the adsorption interactions between graphene and GP species.
- Regarding pore size distribution, both systems exhibited an identical trend of pore size distribution. Notably, at a high probability density of 56.5%, the pore diameters were 1.62 nm for the system with graphene and 1.57 nm for the system without graphene.

## Data Availability

Data are contained within the article.
